# CRISPR/Cas9-Mediated Mutagenesis of *RCO* in *Cardamine hirsuta*

**DOI:** 10.3390/plants9020268

**Published:** 2020-02-18

**Authors:** Claire Lessa Alvim Kamei, Bjorn Pieper, Stefan Laurent, Miltos Tsiantis, Peter Huijser

**Affiliations:** 1 Department of Comparative Development and Genetics, Max Planck Institute for Plant Breeding Research, Carl-von-Linné-Weg 10, 50829 Cologne, Germany; claire.kamei@hrb.bio (C.L.A.K.); pieper@mpipz.mpg.de (B.P.); laurent@mpipz.mpg.de (S.L.); 2 Hudson River Biotechnology, Nieuwe Kanaal 7V, 6709 PA Wageningen, The Netherlands

**Keywords:** CRISPR/Cas9, *Cardamine hirsuta*, *RCO*, leaf development

## Abstract

The small crucifer *Cardamine hirsuta* bears complex leaves divided into leaflets. This is in contrast to its relative, the reference plant *Arabidopsis thaliana*, which has simple leaves. Comparative studies between these species provide attractive opportunities to study the diversification of form. Here, we report on the implementation of the CRISPR/Cas9 genome editing methodology in *C. hirsuta* and with it the generation of novel alleles in the *RCO* gene, which was previously shown to play a major role in the diversification of form between the two species. Thus, genome editing can now be deployed in *C. hirsuta*, thereby increasing its versatility as a model system to study gene function and evolution.

## 1. Introduction

Over the past few years *Cardamine hirsuta*, a small crucifer related to the reference plant *Arabidopsis thaliana*, has emerged as a powerful model system for comparative developmental studies in plants. Both species belong to Lineage I of the Brassicaceae, and parallel genetic studies have provided a powerful platform to identify the molecular causes of trait diversity between these two species and for understanding the morphogenetic basis [1,2,3,4,5,6]. Studies of leaf development in particular have led to major advances in identifying both causal genes and regulatory sequences for the diversification of form and the broader logic through which morphological evolution proceeds [2,7,8,9,10,11]. These efforts were underpinned by the development of essential genetic resources, including genetic and cytogenetic maps, a reference genome sequence, recombinant inbred populations, and of a stable gene transformation protocol by floral dipping [12,13,14]. As genome editing has opened considerable new potential for efforts to connect genotype to phenotype through development and evolution [15], it became important to set up a CRISPR/Cas9 genome editing in this system. To develop and evaluate a protocol for applying CRISPR/Cas9-mediated targeted mutagenesis in *C. hirsuta*, we selected its *RCO* gene as a first target. A previously identified *rco* mutant allele causes an easy to observe leaf phenotype with minimal pleiotropic effects elsewhere in the shoot [2].

## 2. Results

**Figure 1:**
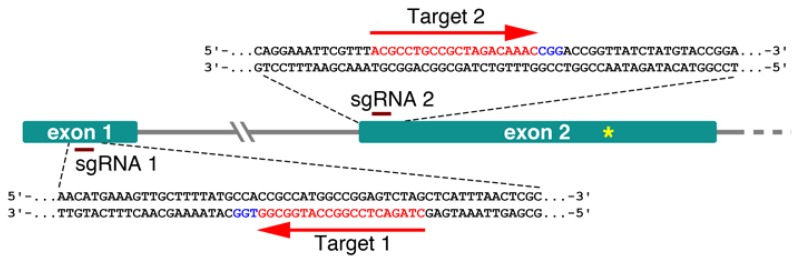
Schematic representation of the *ChRCO* locus targeted for mutagenesis. The two sgRNA complementary sequences are shown in red, and the PAM site is shown in blue. The yellow asterisk in exon 2 marks the position of the premature stop codon causing the first described *rco* mutant allele.

Two sgRNAs were designed to guide the SpCas9 endonuclease to the *RCO* locus in order to generate additional alleles with the potential to uncover new aspects of RCO function (Figure 1).

To ensure the high expression of the SpCas9 protein in Cardamine, we focused on two known strong constitutive promoters: CaMV 35S (p35S) and ubiquitin. The first vector construct carrying the 35S promoter driving the expression of a human codon-optimized Cas9 was assembled using the Golden Gate cloning method [16], with minor modifications. In short, level 1 vectors pICSL11017 (pICH47732::NOSp-BAR-NOST, Addgene no. 51145) and pICH47742::2×35S-5′UTR- hCas9(STOP)-NOST (Addgene no. 49771) and a construct carrying the two sgRNAs, synthesized in tandem (GenScript), each with its own *A. thaliana* U6 RNA polIII promoter, were assembled into the level 2 vector pAGM4723 (Addgene no. 48015). The second construct carrying the ubiquitin promoter has been previously described [17] and takes advantage of the Gateway cloning system. The binary Gateway-compatible destination vector, pDE-Cas9, contains the ubiquitin 4-2 promoter from *Petroselinum crispum* (parsley; pPcUbi4-2) driving the expression of an Arabidopsis codon-optimized *Cas9*. With the help of a Gateway-compatible entry vector, the same two sgRNAs, were transferred into pDE-Cas9 through a Gateway LR reaction. Although both promoters are considered constitutive, neither of them shows uniformly high activity in plants. The CaMV 35S promoter in particular, is weakly expressed in pollen and marginally in embryogenic cells [18]. The position of each sgRNA used in this work is illustrated in Figure 1.

**Figure 2:**
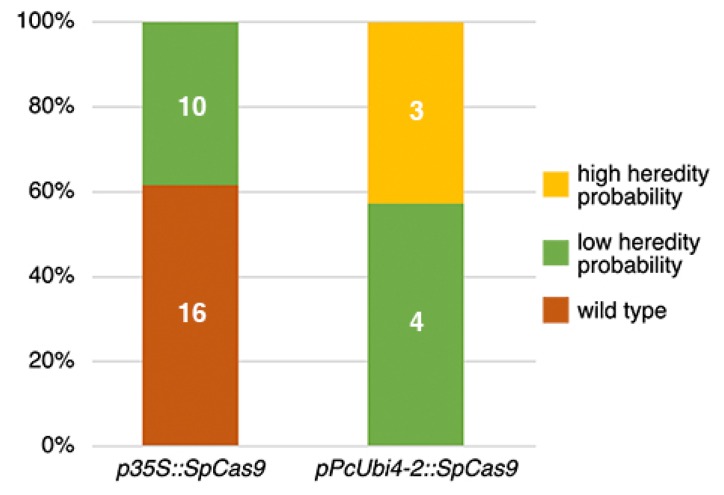
TIDE analysis of T1 Cardamine plants to estimate CRISPR heritable mutations in the sgRNA 1 target site. Plants with 50% or more wild-type sequence traces are classified either as carrying targeted mutations with ‘low heredity probability’, or as ‘wild type’ if indels are present with less than 15%.

Both vector constructs were transformed into *C. hirsuta* Ox by means of the floral dip method [14,19] and their editing efficiency was estimated in the T1 generation using the Tracking of Indels by DEcomposition (TIDE) software [20,21]. To get a first impression of how many heritable mutations could be expected, genomic DNA was extracted from the primary inflorescences of randomly picked T1 plants. Primers to isolate the targeted region were designed following the instructions on the website, and amplified PCR products were further sequenced. The TIDE software compares the sequence trace data from a candidate T1 mutant against the wild type and estimates the indel spectrum and editing efficiency. Candidate mutations were found only for the sgRNA 1 target site, ca. 50 nt downstream of the *RCO* ATG-start codon (Figure 2).

In parallel, the segregation rates of all T1 plants were scored on a selective medium for the presence of the T-DNA-conferred resistance. Only T2 plants with a segregation rate of 3:1 were further analyzed to guarantee the presence of only one copy of Cas9 and thus facilitate the identification of Cas9-free T2 mutants. Of the segregating lines, DNA extracted from the leaves of individual plants was tested by PCR for the absence of Cas9. In the latter case, a second PCR reaction was performed to cover the sgRNA-targeted regions, determine their sequence and analyze using the PolyPeakParser software [22,23] to pinpoint the nature of the generated mutation, e.g., heterozygous, homozygous, or still wild type. A summary of the total number of plants analyzed and their zygosity is presented in Table 1.

**Figure 3:**
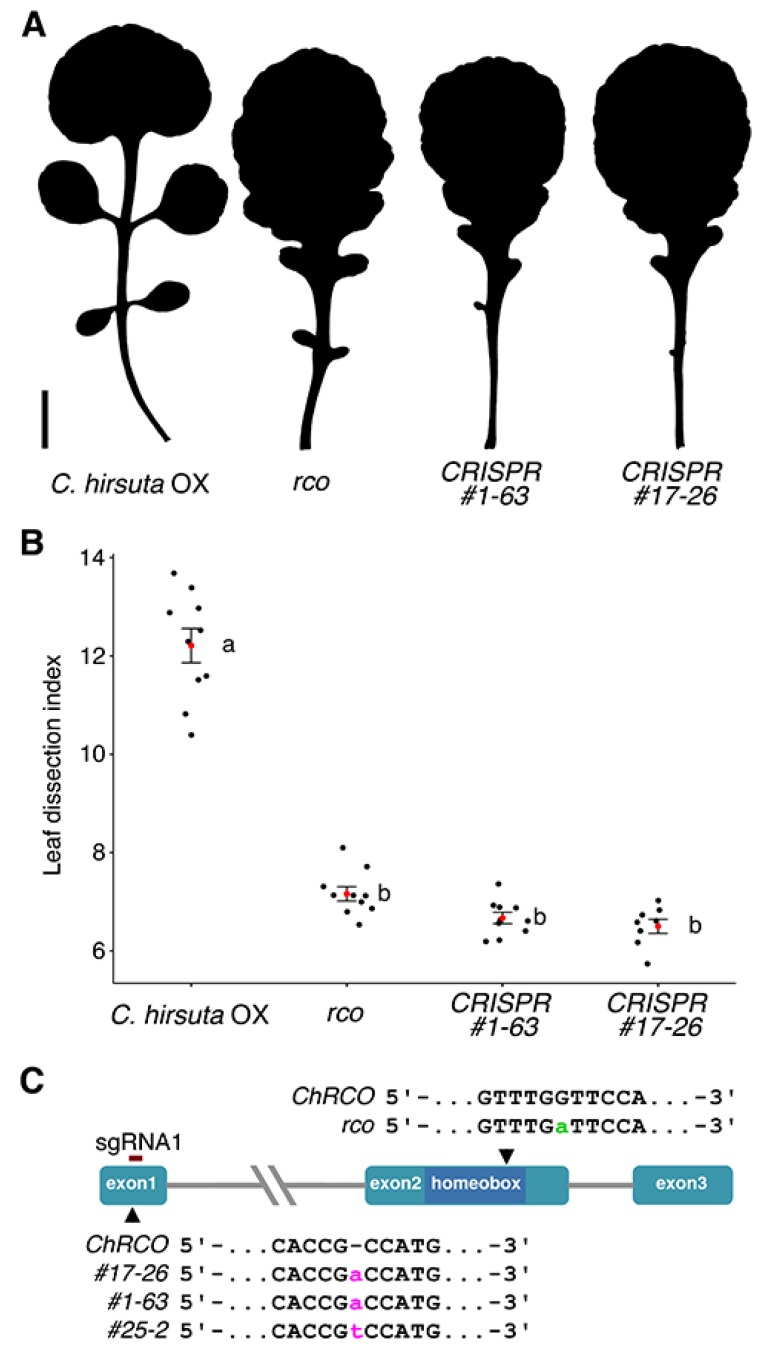
Leaf shape comparison between wild-type and *rco* mutant lines. (**A**) Representative silhouettes of the fifth rosette leaf of the *C. hirsuta* wild type, the original *rco* mutant [2], and of two independently CRISPR-derived *rco* mutant lines. Scale bar: 1 cm; (**B**) Dissection index of the fifth rosette leaf. A red dot indicates the mean and error bars ± 1SD. Significance groups are determined based on ANOVA and Tukey’s HSD test for multiple pairwise comparisons. Only pairwise comparisons involving different groups, labeled a-b, are significantly different below the 5% level; (**C**) a schematic representation of the *RCO* locus. Mutations due to single nucleotide insertions in three independently CRISPR-derived alleles are indicated in lower case magenta. The previously known ‘non-CRISPR’ mutation in the second exon is shown in lower case green.

Phenotypic and genotypic analysis of the Cas9-free homozygous mutant progeny from two independent pPcUbi4-2::Cas9 lines, resp. *#1-63* and *#17-26*, and one p35S::SpCas9 derived line, *#25-2*, is presented in Figure 3. The leaf shape of all three lines (only *#1-63* and *#17-26* shown) clearly differs from the wild type (Figure 3A), e.g., much less dissected (Figure 3B), but is virtually indistinguishable from those of the previously isolated *rco* mutant. Consistent with this, the new CRISPR mutants were found to be allelic to the original *rco* mutant allele in an allelism test.

To check for the presence of possible off-target mutations, whole genomes of the three respective mutants were sequenced in depth and compared against the *C. hirsuta* Ox reference. For both guide RNAs, no mutations (Indels or SNPs) were found in four off-target intervals, including *ChLMI1-LIKE3* (CARHR209480) predicted by Cas-OFFinder9 (v2.4) [24,25], with an edit distance of up to 4 bp. Within the intended location in the *RCO* gene (CARHR209490) an insertion of an A was found in samples *#1-63* and *#17-26* and an insertion of a T in *#25-2* as depicted in Figure 3C. As expected, the respective genotypes lacked the T-DNA insertion containing Cas9, were homozygous for the mutant alleles and the insertions located within the sgRNA 1 target site, 4 nt upstream of the PAM site (Figure 1).

## 3. Discussion

**Figure 4:**
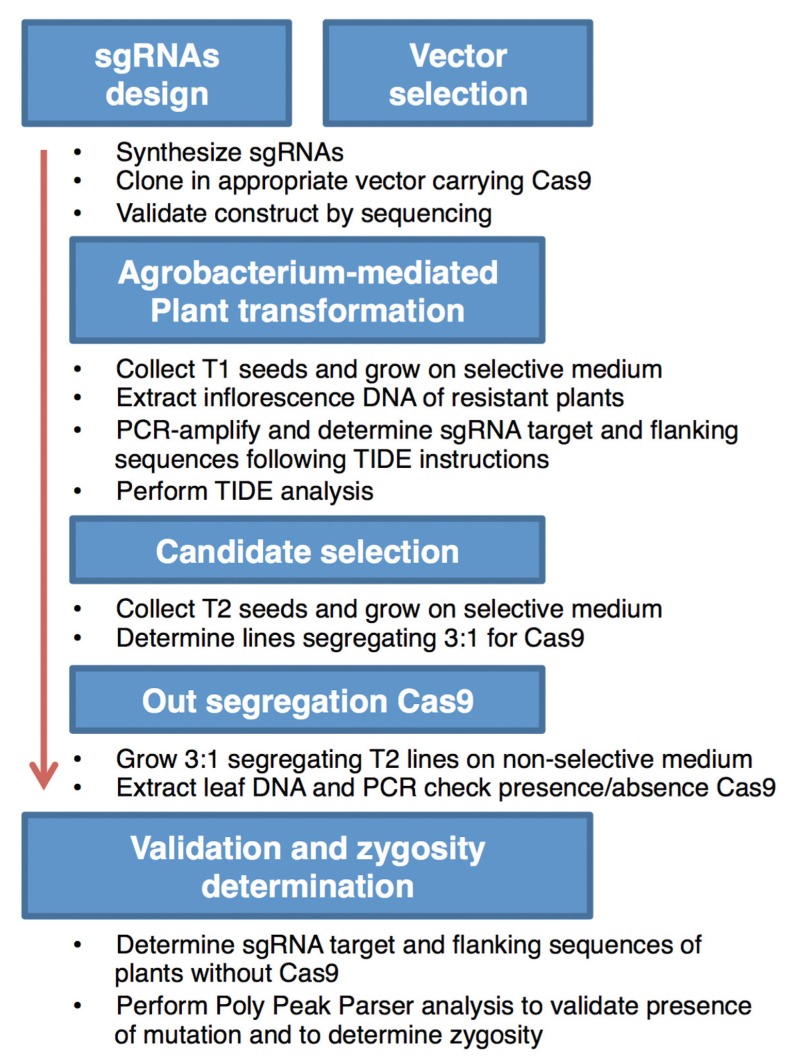
Simple flow diagram of the CRISPR/Cas9-mediated mutagenesis process, as followed in *C. hirsuta* in order to generate novel *rco* mutant alleles.

Using two available and previously described CRISPR/Cas9 expression constructs and following the protocol, as depicted in Figure 4, we successfully generated targeted mutations in the form of 1 bp insertions in the *RCO* locus of *C. hirsuta*.

For the construct carrying p35S::SpCas9, such mutations could be detected only in ca. 40% of the T1 plants which, moreover, all had low probability of heredity, i.e., present with low frequency in reproductive tissue. In this respect, the Arabidopsis codon-optimized Cas9 driven by the parsley PcUbi4-2 promoter clearly performed better. For the latter, all tested T1 plants carried targeted mutations with ca. 60% of these with low and ca. 40% with a high probability of heredity. The 0.2% versus the 3.8% homozygous and Cas9 free mutant plants found in the T2 generation supported this difference in efficiency and heredity.

Despite the relatively low number of 33 T1 plants tested for both constructs in total, it is noteworthy that targeted mutations could be detected for sgRNA 1 only. The reason for this difference in sgRNA efficiency is unclear, but it endorses the general advice to design and test multiple sgRNAs to maximize the chance of mutating a target gene. In conclusion, we provided a workflow for genome editing that will increase the versatility of *C. hirsuta* as a model for comparative studies as well as additional alleles of *rco* which will help further dissection of genetic pathways determining leaf shape.

## 4. Materials and Methods

### 4.1. Plant Material, Vector Constructs, and Cardamine hirsuta Transformation

*C. hirsuta* plants, Oxford (Ox) accession, were grown in long day conditions (16 h light) in the greenhouse. The origin of the plasmid vectors used, the design and synthesis of the sgRNAs, and their assembly using either the Golden Gate or Gateway cloning method has been described with the appropriate references in Section 3 of the main text. All vectors were transformed into *C. hirsuta* Ox by *Agrobacterium tumefaciens* (strain GV3101)-mediated floral dip, as previously described [14,19].

### 4.2. Primers Used for PCR Analysis

Primer pairs (forward/reverse) used to PCR-amplify sgRNA target regions:

ChRCO_sgRNA1 TCGTATGGTCCAACAAAACC/GTGAAATTTAACGGCCACTTAC

ChRCO_sgRNA2 GTGATTCTTCCGCTTTCTCTTC/GAACCAAACCGCTACCTGAC

For TIDE analysis of T1 plants:

TIDE_sgRNA1 CACCTCCAAAAGACAGTATAGAAGA/TAACTGCTGCCTAGGAATGTTT

TIDE_sgRNA2 GTTTTTCTTGGGAACCAGCTC/CTCTCAGCTTATTCACCTGCATTAT

To check presence/absence of *Cas9*:

T2Gateway GTATGTATATATGTAGATCTGG/GAAGTTAGACTTGAAGTTAGG

T2GoldenGate CTAGATCGACGCTTGCTGAA/TCGGTATTGCCCAGAACTTT

### 4.3. Leaf Shape Phenotyping

Digitized leaf silhouettes of developed rosette leaves were obtained with the help of a flatbed scanner. The leaf silhouette area (A) and perimeter (P) were determined within FIJI [26] and used to calculate the leaf dissection index, P/sqrt(A).

### 4.4. Genome Sequencing and Off-Target Analysis

Genome DNA sequencing was performed by the Max Planck-Genome-centre Cologne, Germany (https://mpgc.mpipz.mpg.de/home/), on an Illumina HiSeq3000, and the data quality of fastq files was assessed using FastQC (v0.11.7) [27]. Multiple fastq files for each individual sample were combined, and clumpify.sh from the BBMap (v38.34) package [28] was used to remove duplicates before mapping. Trim_galore (v0.4.3) [29] was used to quality trim and remove adapters. Mapping was performed using bwa (v0.7.17-r1188) [30]. Picard (v2.20.8) (http://broadinstitute.github.io/picard/) was used to mark optical duplicates. Indel realignment was performed using the Genome Analysis Toolkit GATK (v3.8-0-ge9d806836) [31] and SNP and Indel calling done with VarScan (v2.4.2) [32]. The pileup of reads, which is an input for VarScan, was generated using samtools (v1.9) [33]. The resulting VCF (Variant Call Format) file for Indels was run through bcftools norm (v1.9) [34] to split multiallelic sites in separate entries. The off-target locations were determined using an offline version of Cas-OFFinder (v2.4) [25] with an edit distance of up to 4 bp. Bcftools (v1.9) was used to check for overlaps of entries in VCF files with off-target locations. The variants in the VCF files were annotated using snpEff (v4.4) [35]. The database for snpEff annotation was created from the annotation file available for *C. hirsuta* found at http://chi.mpipz.mpg.de/download/annotations/carhr38.gff. A fasta file with the reference *C. hirsuta* genome sequence can be found at http://chi.mpipz.mpg.de/download/sequences/chi_v1.fa.

## Figures and Tables

**Table 1:** T2 analysis to identify homozygous CRISPR mutants lacking Cas9.

Construct	Segregating Lines Identified	Plants Screened	Cas9 Free Homozygous Mutants
*p35S::SpCas9*	5	478	1 (0.2%)
*pPcUbi4-2::SpCas9*	2	159	6 (3.8%)
